# Biocontrol Potential of *Bacillus amyloliquefaciens* against *Botrytis pelargonii* and *Alternaria alternata* on *Capsicum annuum*

**DOI:** 10.3390/jof7060472

**Published:** 2021-06-10

**Authors:** Elham Ahmed Kazerooni, Sajeewa S. N. Maharachchikumbura, Abdullah Mohammed Al-Sadi, Sang-Mo Kang, Byung-Wook Yun, In-Jung Lee

**Affiliations:** 1 Department of Applied Biosciences, Kyungpook National University, Daegu 41566, Korea; rhizobacteria@gmail.com (S.-M.K.); bwyun@knu.ac.kr (B.-W.Y.); 2 School of Life Science and Technology, University of Electronic Science and Technology of China, Chengdu 611731, China; sajeewa83@yahoo.com; 3 Department of Plant Sciences, College of Agricultural and Marine Sciences, Sultan Qaboos University, Al-Khod 123, Oman; alsadi@squ.edu.om

**Keywords:** plant growth promoting rhizobacteria, antagonism, disease suppression, pepper, hydrolytic enzymes

## Abstract

The aim of this study was to assess the ability of *Bacillus amyloliquefaciens*, to augment plant growth and suppress gray mold and leaf spot in pepper plants. Morphological modifications in fungal pathogen hyphae that expanded toward the PGPR colonies were detected via scanning electron microscope. Furthermore, preliminary screening showed that PGPR could produce various hydrolytic enzymes in its media. Treatments with *B. amyloliquefaciens* suppressed Botrytis gray mold and Alternaria leaf spot diseases on pepper caused by *Botrytis pelargonii* and *Alternaria alternata*, respectively. The PGPR strain modulated plant physio-biochemical processes. The inoculation of pepper with PGPR decreased protein, amino acid, antioxidant, hydrogen peroxide, lipid peroxidation, and abscisic acid levels but increased salicylic acid and sugar levels compared to those of uninoculated plants, indicating a mitigation of the adverse effects of biotic stress. Moreover, gene expression studies confirmed physio-biochemical findings. PGPR inoculation led to increased expression of the CaXTH genes and decreased expression of CaAMP1, CaPR1, CaDEF1, CaWRKY2, CaBI-1, CaASRF1, CaSBP11, and CaBiP genes. Considering its beneficial effects, the inoculation of *B. amyloliquefaciens* can be proposed as an eco-friendly alternative to synthetic chemical fungicides.

## 1. Introduction

Pepper (*Capsicum annuum* L., family Solanaceae) is one of the most economically important vegetable crops and consumed spices around the world. Pepper fruits can be consumed in fresh, dry, and processed forms and numerous health benefits are associated with their consumption. They are a rich source of vitamins, minerals, and antioxidants and help to hinder inflammation, cancer, and cell damage and improve the immune system. Their compounds are used in commercial medicinal products to treat muscle pains, arthritis, stomach ulcers, etc. [1]. Pepper consumption has surged in the last 20 years [2] and further increases are expected due to higher demand by consumers. However, this crop is highly susceptible to a broad range of pests and diseases; thus, plant yield and productivity are affected by such stresses, leading to economic losses [3].

*Botrytis* and *Alternaria* spp. attack a wide range of crops, including more than 1000 species of vascular plants. Geographically, they occur wherever their host plants grow, ranging from extreme cold areas to hot desert regions. They trigger diseases on all parts of the plant, including the seed, flower, fruit, leaf, and shoot, and impose serious damage to agricultural crops [4,5,6,7,8]. Alternaria and Botrytis spp. are common pathogens of *Capsicum* spp. worldwide. In Malaysia, *Alternaria capsicicola* is a causal agent of leaf spot of *C. annum* [9], while *A. alternata* is the common pathogen of *C. annum* leaf spot [10]. On the other hand, *Botrytis cinerea* is the dominant causal agent of *Capsicum* gray mold in different parts of the world [11,12,13,14,15]. *Botrytis pelargonii* has also been reported associated with gray mold in some crops [16].

Despite fungicides being beneficial for controlling different diseases, including *Capsicum* leaf spot and gray mold, their intensive use is deleterious to the surrounding environment and to the existence and sustainability of beneficial rhizosphere microbes [17,18]. Likewise, the rising price of pesticides as well as consumer demand for pesticide-free food has resulted in the exploration of alternatives for these products. Consequently, there is a demand to identify effective substitutes to environmentally degrading synthetic pesticides.

Rhizobacteria that benefit plants by inducing growth and restraining disease are known as plant growth-promoting rhizobacteria (PGPR) [19]. PGPR have been examined as biocontrol agents for the repression of plant diseases [20] and, additionally, as stimulators of disease endurance in plants [21,22]. In particular, strains of *Pseudomonas*, *Stenotrophomonas*, and *Bacillus* have been effectively applied to manage plant pathogens and increase plant growth [21,23,24]. The extensively distinguished mechanisms of plant growth promotion by PGPR are phytohormone production, nitrogen fixation, and phosphate solubilization. Mechanisms of biocontrol activity comprise competition with plant pathogens for an environmental niche or nutritional resources along with the production of hydrolytic enzymes and antimicrobial compounds that are usually active against a wide range of plant pathogens [25,26,27,28].

There is potential for the application of microbial antagonists for the management of *Botrytis* and *Alternaria* spp. on crops [6]. Filamentous fungi, such as *Trichoderma* and *Gliocladium* spp., and bacteria, such as *Bacillus* and *Pseudomonas* spp., have exhibited great capabilities for *Botrytis* spp. disease management [29]. Ramírez-Cariño et al. [30] demonstrated successful control of *A. alternata* and *Fusarium oxysporum* through the use of *Bacillus paralicheniformis* and *Trichoderma asperelloides* on tomato plants. Few studies have addressed the biological control of *Alternaria* and *Botrytis* spp. in *Capsicum* spp. Bacterial isolates obtained from compost have shown efficacy in inhibiting *Botrytis* gray mold and *Alternaria* fruit rot in *Capsicum* spp. [13]. Sid, Ezziyyani, Egea-Gilabert, and Candela [10] showed that isolates belonging to *Bacillus* spp. reduced Alternaria leaf spot and increased the dry mass of *Capsicum* plants.

Previous studies showed that *Bacillus amyloliquefaciens* could suppress the growth of *Aspergillus parasiticus*, *Phytophthora capsici*, *Fusarium oxysporum*, *Botryosphaeria dothidea* and promote the growth of plant [31,32,33]. The objectives of the present study were to investigate the ability of *B. amyloliquefaciens* to promote *Capsicum* spp. growth and suppress *Botrytis* gray mold and *Alternaria* leaf spot. The PGPR (*B. amyloliquefaciens*) was isolated from *Sasamorpha borealis* and the following PGP traits: indole acetic acid production; nitrogen fixation; 1-aminocyclopropane-1-carboxylate deaminase activity; siderophore production; citrate utilization; inorganic phosphate, potassium, zinc, and silicon solubilization were characterized in this strain [34]. This research work will assist with the understanding of whether *B. amyloliquefaciens* has suppressive effects on a variety of fungal pathogens and whether it promotes activities on different crops.

## 2. Materials and Methods

### 2.1. Collection Site and Isolation of Fungal Pathogens

Pepper plants (*C. annuum* cv. Geumsugangsan) with symptoms of disease were harvested from the pepper agricultural farm situated at Kyungpook National University (Gunwi-gun), Daegu, Korea (36°06′48.5″ N 128°38′26.4″ E), employing organic cultivation practices (Figure S1). Samples were immediately placed into Ziplock bags, transported to the laboratory, and stored at 4 °C. Fungal pathogens were isolated from the infected parts of the plants as described by Romero et al. [35] and kept on potato dextrose agar (PDA) medium at 25 °C for further analysis. The PGPR (*B. amyloliquefaciens*) was obtained from our previous study (accession no. MW599955).

### 2.2. Molecular Characterization of Fungal Isolates

Genomic DNA was obtained from fresh fungal cultures (seven days old) as per the methods of Al-Sadi et al. [36]. Amplification reactions were performed using the BioFACT™ 2X Multi-Star PCR Master Mix (BIOFACT, Daejeon, Korea) and a combination of primers (ITS1/ITS4, RPB2-5F2/fRPB2-7cR, Alt-for/Alt-rev, and gpd 1/gpd 2) according to the defined conditions [37,38,39] (Table S1). PCR products were purified and sequenced at SolGent Co., Ltd. (Daejeon, Korea). Sequence data for the ITS, GAPDH, and Alt a 1 were obtained for the isolate of *Alternaria* (ALT), which resulted in the GenBank accession numbers, MW793507, MW803061, and MW803062, respectively. The RPB2 gene sequences obtained for *Botrytis* isolate (BOT) were logged in the GenBank database under accession number MW803063. Two different datasets were used to estimate two phylogenies: a species tree in *Alternaria* section Alternaria based on combined ITS, GAPDH, and Alt a 1 gene and a *Botrytis* species tree based on RPB2 gene region. The phylogenetic analyses were performed using the raxml GUI v1.3 [40] and the dendrogram was created with MEGA v7.0.26 software.

### 2.3. Pathogenicity of Fungal Isolates in Pepper Seedlings

The pathogenicity test of isolated fungi was carried out on the pepper cultivar Geumsugangsan (Takii Korea Ltd., Seoul, Korea). Inoculation with fungal pathogens (ALT and BOT) was conducted by pipetting individual droplets of fungal suspension on the surface of healthy leaves. Control plants were treated with sterile distilled water (SDW) only. Inoculated plants were maintained in a humid chamber (250 μmol photons m^−2^ s^−1^ PAR, 70% relative humidity, 25 ± 2 °C, 16:8 h light:dark cycle). After symptoms appeared on the seedlings, the fungus was re-isolated from symptomatic tissues and its identity was confirmed by morphological and molecular studies.

### 2.4. In Vitro Evaluation of the Antifungal Activity of the Bacterial Strain Against Fungal Pathogens

The ability of the bacterial strain to antagonize ALT and BOT isolates was examined via a dual culture method [41]. The inoculated plates were kept at 28 ± 2 °C until the leading edge of the fungus in the control plate reached the edge of the plate. The antagonistic effect of the bacterial strain against ALT and BOT was confirmed by the formation of an inhibition zone. Moreover, the effect of the chosen bacterial strain on the hyphal morphology of ALT and BOT was visualized using a scanning electron microscope (SEM; Hitachi SU8220, Tokyo, Japan). The SEM sample preparation was carried out as defined by Heckman et al. [42].

### 2.5. Determination of Hydrolytic Enzyme Activity of the Bacterial Strain

The ability of the bacterial strain to produce amylase, protease, pectinase, and cellulase was determined [43]. The catalase activity test was conducted by adding three-to-four drops of hydrogen peroxide (H_2_O_2_) to the bacterial culture, which was grown on trypticase soy agar medium. The effervescence confirmed the catalase activity of the bacterial strain [44]. To determine phytase activity, bacterial strain was inoculated onto medium containing sodium phytate [45]. Qualitative lipase activity was evaluated on Tween agar medium. The appearance of a white precipitate indicated positive lipolytic activity [46,47]. The activity of laccase was detected in the medium supplemented with gallic acid. The formation of a dark brown color around the colony was the result of laccase activity [48]. The method of Balasubramanian et al. [49] was used for the visual detection of glucanase activity of the bacterial strain.

### 2.6. In Vivo Evaluation of the Antifungal Activity of the PGPR against ALT and BOT

#### Preparation of the Fungal and Bacterial Inocula

The fungal inoculum was established as described previously [50]. Conidia were suspended in a solution containing glucose and potassium phosphate (10 mM, pH 6) to stimulate the infection on pepper leaves.

The chosen bacterial strain was used to prepare the bacterial inoculum suspension at the optical density (600 nm). The bacterial strain was cultured in lysogeny broth (120 rpm, 24 h, 28 ± 2 °C) and bacterial cells were collected via centrifugation (5000 rpm, 10 min, 4 °C). The obtained pellet was washed three times with SDW. Afterward, the obtained cell pellets were suspended in 0.03 M MgSO_4_ (10^8^–10^9^ CFU/mL), vortexed, and used for plant treatment (50 mL/pot).

### 2.7. Plant Material and Growing Conditions

Seeds of *C. annuum* cv. Geumsugangsan were disinfected by washing with 70% ethanol (1 min) and 1.5% sodium hypochlorite (5 min) followed by rinsing three times with SDW. Thereafter, the disinfected seeds were evaluated for efficiency of the sterilization process [51] and viability [52]. Pre-germinated seeds were placed in sterilized plastic pot trays (28 × 54 cm) containing autoclaved sterilized soil (Shinsung Mineral Co., Ltd., Chungcheongbuk-do, Korea). One seed was sown in each pot. Seeds were grown for three weeks in a humid chamber as described above and watered daily.

### 2.8. Experimental Design

Various treatments were applied to three-week-old seedlings two days after transplanting. The seedlings were split into the two following groups: the normal control group irrigated with SDW (50 mL/pot) and the PGPR group irrigated with bacterial inoculum suspension (50 mL/pot). Each group was treated for seven days, after which the seedlings with or without PGPR treatment were further split into two groups with an equal number of seedlings. This formed six experimental groups, which are described in Table 1. The pepper seedlings were exposed to selected biotic stresses, and sampling was performed after eight days [53,54]. The harvested samples were either immediately used or rapidly deactivated in liquid nitrogen and stored at −80 °C.

### 2.9. Determination of Soil Moisture, pH, and Electrical Conductivity (EC)

The moisture level (70%), pH value (~7), and EC (≤1.2) of the bulk soil samples were recorded before the pot experiment. The soil moisture level of each pot was monitored daily using a humidity tester (Model DM-5, Takemura Electric Works, LTD., Tokyo, Japan). Furthermore, at harvesting, a soil sample from certain pots per treatment was used to determine pH, EC, and moisture using the humidity tester and conductivity meter (YSI Model 32, Yellow Spring, OH, USA) (Table S2).

### 2.10. Physio-Biochemical Attributes of the Pepper Plant

#### Plant Growth Characteristics and Photosynthetic Pigments

To assess the effect of each treatment on the seedlings, multiple plant growth parameters were evaluated. These parameters comprised plant height, stem diameter, leaf area (length/width), total plant fresh weight, and number of leaves, which were recorded after eight days. A digital Vernier caliper and a ruler were used to measure the stem diameter, leaf area (length and width), and plant height.

Leaf chlorophyll a (Chla), chlorophyll b (Chlb), total chlorophyll, and carotenoid contents were determined by the spectrophotometric analysis of chemically extracted pigments [55]. Briefly, the freeze-dried ground leaves were extracted in 80% ethanol at room temperature after centrifugation. Pigment absorption was measured spectrophotometrically at 663, 645, and 480 nm (Thermo Fisher Scientific, Waltham, MA, USA).

### 2.11. Phytohormone Analysis; Abscisic acid (ABA) and Salicylic Acid (SA)

The pepper ABA content was extracted and analyzed according to the previously described method [56]. Nitrogen gas (N_2_) was added to dry out the resultant extract, and its methylation was achieved using diazomethane (CH_2_N_2_). ABA content was quantified by GC-MS (Agilent 6890N Gas Chromatograph, Santa Clara, CA, USA). ThermoQuest software (Manchester, UK) was used to observe the responses to ions (m/e of 162 and 190 for Me-ABA and 166 and 194 for Me-[^2^H_6_]-ABA).

The level of SA in the pepper plants was estimated as outlined previously [57,58]. Briefly, the freeze-dried sample (0.1 g) was extracted with methanol (90% and 100%) by centrifugation (12,000 rpm for 15 min at 4 °C). The combined methanol extracts were then vacuum-dried. The dried residue was dissolved in 5% trichloroacetic acid and centrifuged at 10,000 rpm for 10 min. The supernatant was partitioned with ethyl acetate/cyclopentane/isopropanol (49.5:49.5:1, *v*/*v*). The top layer of the aqueous solution was dried and used for SA quantification using high-performance liquid chromatography.

### 2.12. Amino Acid Content of the Leaves

The amino acid content was determined by hydrolyzing freeze-dried leaves (50 mg) in 1 mL of hydrochloric acid (6N HCl) for 24 h at 110 °C [59]. Then, the extraction was condensed and dried with a vacuum at 80 °C for 24 h. Afterward, the residue was diluted with deionized water (2 mL) and evaporated twice. Finally, the concentrated residue was dissolved with hydrochloric acid (0.02 N HCl, 1 mL) and the mixture was passed through a 0.45 μM filter membrane. The solution was analyzed using a Hitachi L-8900 Amino Acid Analyzer (Hitachi High-Technologies Corporation, Tokyo, Japan).

### 2.13. Estimation of the Leaf Protein and Sugar Content

The soluble protein content subjected to different treatments was quantified following the methods of Ashraf and Iram [60], and bovine serum albumin was used as a standard. The leaf samples (0.1 g) were crushed and later blended with 1 mL of phosphate buffer (50 mM, pH 7.0). The mixture was then centrifuged at 10,000 rpm for 10 min at 4 °C. Afterward, the appropriate reagent was added to the obtained supernatant and the absorbance of each sample was recorded at 595 nm.

The total sugar content was determined as described by Khan et al. [61]. Specifically, freeze-dried leaves were ground and extracted with 80% ethanol followed by vacuum drying. The dried remnant was re-dissolved in 1 mL of deionized water and passed through 0.45 μM Nylon-66 syringe filters. Furthermore, the filtered samples were injected into a high-performance liquid chromatograph (Millipore Co., Waters Chromatography, Milford, MA, USA).

### 2.14. Enzymatic and Nonenzymatic Antioxidant Activity

The antioxidant enzyme assays for peroxidase (POD) and polyphenol oxidase (PPO) were performed using the method of Putter [62]. Superoxide dismutase (SOD) content was analyzed by the method proposed by Sirhindi et al. [63]. To determine flavonoid content, DPPH radical scavenging activities, and total polyphenol samples, were processed following previously described procedures [64,65,66,67]. The mixture activity and absorbance were measured at selected wavelengths using the Multiskan™ GO UV/Vis microplate spectrophotometer (Thermo Fisher Scientific, Waltham, MA, USA).

### 2.15. Hydrogen Peroxide and Lipid Peroxidation (Malondialdehyde (MDA)) Contents

The H_2_O_2_ level of plants subjected to various treatments was determined as per the methods described previously [68,69]. The frozen samples were freeze-dried then ground finely. The powdered sample (0.3 g) was homogenized with 3 mL of ice-cold phosphate buffer (50 mM, 1 mM EDTA, 1% PVP, pH 7.0) and centrifuged at 13,000 rpm for 20 min. The supernatant (2 mL) was blended with 1 mL of 20% (*v*/*v*) H_2_SO_4_ containing 0.1% titanium chloride and the mixture was then centrifuged at 13,000 rpm for 20 min. The supernatant intensity was measured at 410 nm with a T60 UV-Vis Spectrophotometer (PG instruments Ltd., Wibtoft, UK).

The method of López-Serrano et al. [70] was employed to estimate the amount of lipid peroxidation. The MDA content was calculated using its extinction coefficient. The lipid peroxidation content was expressed as the level of MDA created per gram of tissue.

#### Quantification of the Nutrient Content in Pepper Plants

To examine the nutrient contents of the pepper plants, samples were freeze-dried and processed into a powder. Eventually, the prepared samples were subjected to the quantification of nutrient (potassium, K; phosphorus, P; calcium, Ca) uptake in pepper plants using ICP-MS (Optima 7900DV, Perkin-Elmer, Akron, OH, USA). Treatments without bacterial inoculation were performed to determine the initial concentration of the nutrients.

### 2.16. cDNA Synthesis and Real-Time PCR Analysis

Total RNA was extracted from the pepper leaves harvested at the end of the experiment. The obtained RNA was employed for cDNA synthesis and quantitative PCR following the previously described procedure [71]. Specifically, 1 μg of RNA was consumed to synthesize cDNA using the BioFACT^TM^ RT-Kit (BIOFACT, Daejeon, Korea) following the manufacturer’s standard protocol. The synthesized cDNA was used as a pattern in a two-step qRT-PCR reaction carried out to determine the transcript quantity with an Illumina Eco^TM^ system (Illumina, San Diego, CA, USA) (Table S3).

### 2.17. Data Analysis

Statistical analysis was performed using R software (v4.0.3) and Microsoft Excel 2017. Treatments were compared via analysis of variance using the least significant difference test at a 5% probability level (*p* < 0.05). The graphs were prepared using GraphPad Prism software (v6.01, San Diego, CA, USA). A completely randomized design was applied for all experiments, with three replications and three repetitions.

## 3. Results

### 3.1. Identification of Fungal Isolates

**Figure 1:**
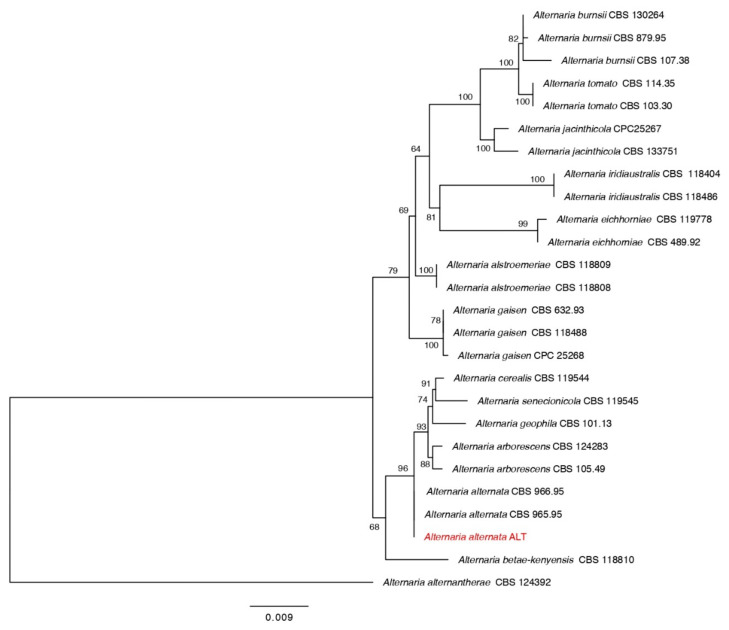
Maximum likelihood tree obtained from the combined ITS, GAPDH, and Alt a 1 sequence alignment analysis of the species in section *Alternaria*. Bootstrap values (>50) are represented by numbers at the nodes based on 1000 replications. The strain in red font is from our study.

**Figure 2:**
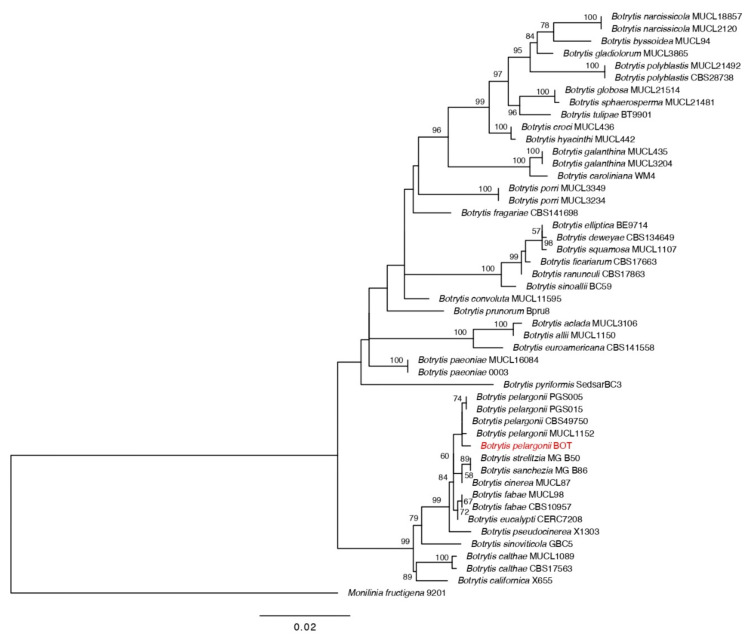
Maximum likelihood tree based on RPB2 nucleotide sequences for the selected isolates of species in the genus *Botrytis*. Bootstrap values (>50) are represented by numbers at the nodes based on 1000 replications. The strain in red font is from our study.

Two pathogenic fungi were isolated from diseased pepper plants. The phylogenetic analysis based on combined sequences of ITS, GAPDH, and Alt a 1 strongly supported our *Alternaria* isolates to be the *Alternaria alternata* (Figure 1). Further, results of the RPB2 tree for the genus *Botrytis* demonstrated that the isolate BOT formed a strongly supported clade with the *Botrytis pelargonii* (Figure 2).

### 3.2. Bacillus amyloliquefaciens Antifungal Hydrolytic Enzyme Activity and Effect on the Morphology of B. pelargonii and A. alternata

**Figure 3:**
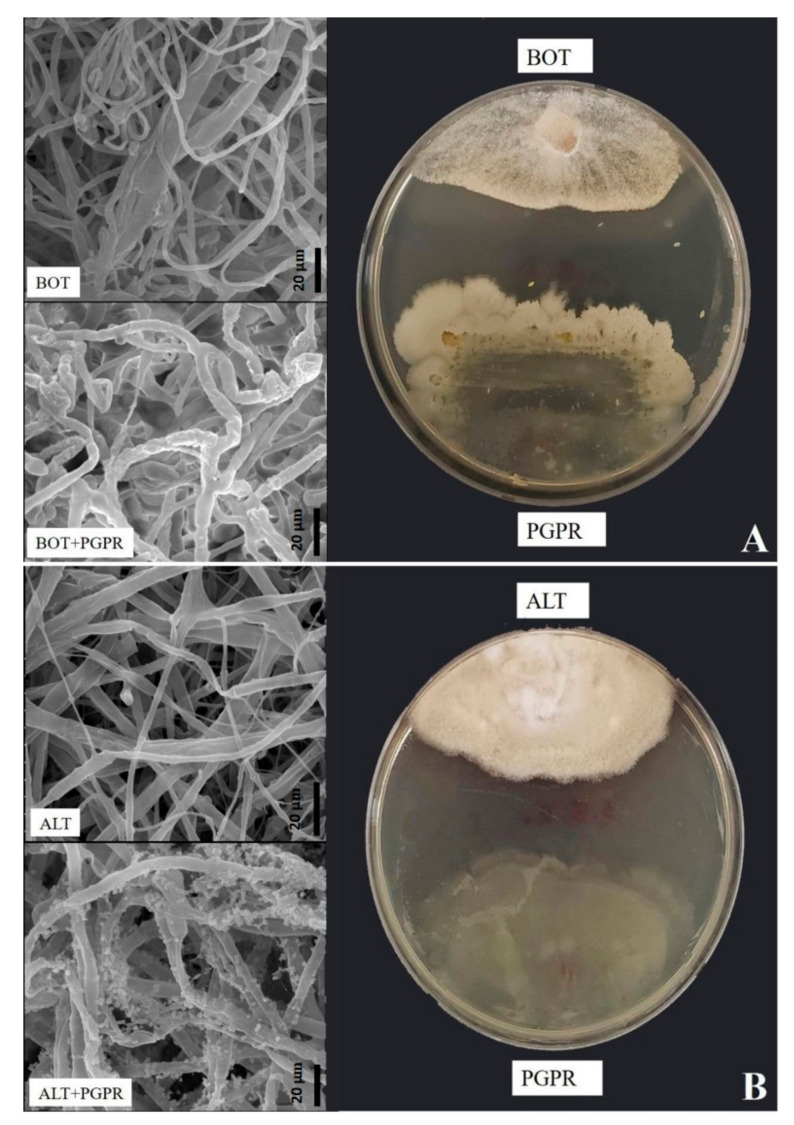
Effect of *Bacillus amyloliquefaciens* on *Botrytis pelargonii* and *Alternaria alternata* hyphae morphology evidentiated using a scanning electron microscope. (**A**,**B**) Abnormal hyphae. Wrinkled, ruptured, or shrunken patterns under *Bacillus amyloliquefaciens* treatment. (**A**,**B**) Normal patterns of hypha in the control.

**Figure 4:**
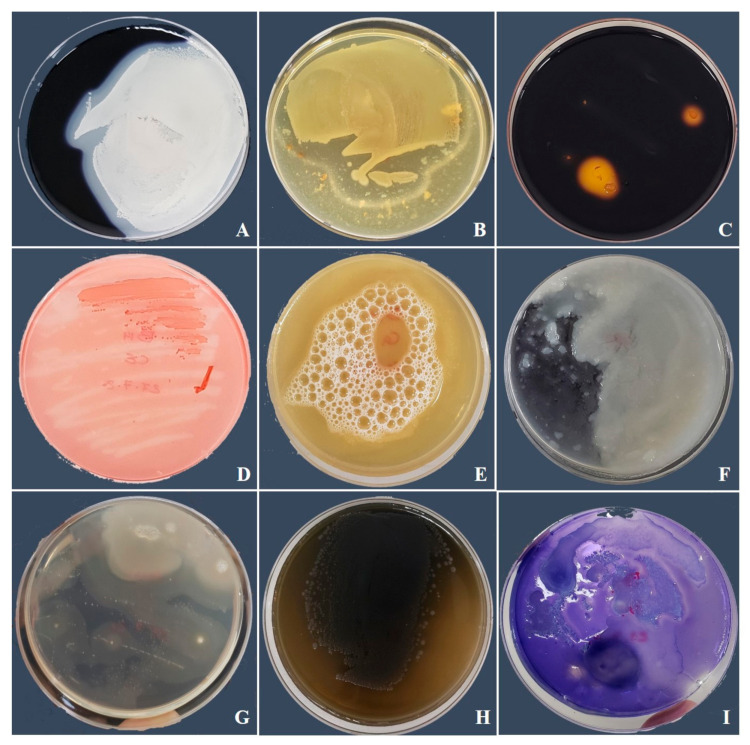
Hydrolytic enzyme activity of bacterial strains in this study. (**A**) Amylase, (**B**) protease, (**C**) pectinase, (**D**) cellulase, (**E**) catalase, (**F**) phytase, (**G**) lipase, (**H**) laccase, and (**I**) glucanase.

The antagonism test showed substantial suppression of *B. pelargonii* and *A. alternata* in the PDA plate under the influence of *B. amyloliquefaciens*. This suppression was demonstrated by the production of an inhibition zone (Table 2). Scanning electron microscopy examination showed that *B. amyloliquefaciens* induced notable changes in the general appearance of *B. pelargonii* and *A. alternata* hyphae. The morphology of both fungal hyphae was modified after being exposed to *B. amyloliquefaciens*. As shown in Figure 3A,B, the hyphae of these fungi were irregular, ruptured, wrinkled, and deformed compared to those of the control. In terms of hydrolytic enzyme activity, *B. amyloliquefaciens* was positive for all examined hydrolytic enzymes (Figure 4, Table 2).

### 3.3. Pepper Seedling Response to B. amyloliquefaciens Inoculant under Biotic Stress

#### Soil Properties

Soils from different treatments were examined to evaluate the impact of PGPR and pathogen on the soil moisture level, pH value, and EC content. The pH value of all soil samples was found to be alkaline (pH = 6.9–7.9), while the EC content ranged from 0.3 to 2.9 mS (Table S2). The EC content was slightly higher in PGPR-inoculated plants subjected to non-stress and stress conditions. The level of moisture was significantly higher in PGPR treated plants with or without biotic stresses.

### 3.4. Impact of PGPR on Plant Growth Attributes

The impact of the PGPR strain on the growth promotion of the pepper seedlings under no stress as well as biotic stress conditions was evaluated through pot trials (Figure S2A,B). The unfavorable consequences of pathogen invasion led to the reduction in growth parameters; namely, plant height, stem diameter, leaf area (length/width), total plant fresh weight, and the number of leaves of the pepper plants, in comparison with non-diseased, un-inoculated pepper plants (Table 3). In contrast, the application of PGPR increased plant height, stem diameter, leaf area (length/width), and total fresh weight in the inoculated plants exposed to pathogen stress. The plant height improved by 32.40% in the *Botrytis* treatment group and by 18.13% in the *Alternaria* treatment group compared to those of the corresponding un-inoculated stressed plants (*p* < 0.05). Similarly, in PGPR-treated plants, the total plant fresh weight increased by 34.83% and 26.22% in *Botrytis* and *Alternaria* treatment groups, respectively, compared to those of the control group of stressed plants (Table 4).

### 3.5. Chlorophyll and Carotenoid Contents

The chlorophyll and carotenoid contents were determined for pepper plants under both normal and stressed conditions. Biotic stresses adversely influenced the photosynthetic pigments of pepper plants. The analysis of plant pigments showed that the Chla/Chlb and carotenoid levels increased in infected plants inoculated with the bacterial strain compared to those that were infected but not inoculated. Likewise, all the PGPR-treated infected plants showed higher total chlorophyll levels than those of the control infected plants (Table 3). Decreases of 9.96% and 40.34% in total chlorophyll content were observed in *Botrytis* and *Alternaria*-stressed plants compared to those of control plants. PGPR inoculation was effective (*p* < 0.05) and caused approximately 31.07% and 57.88% increases under *Botrytis* and *Alternaria* stress conditions compared to those of control infected plants, respectively (Table 3).

### 3.6. Phytohormones; ABA and SA Accumulation

**Figure 5:**
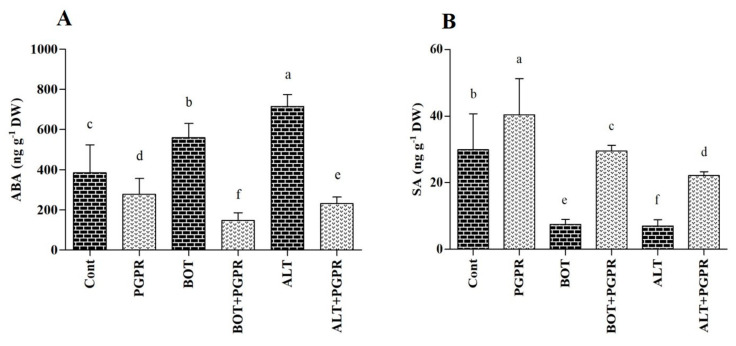
(**A**) Abscisic acid and (**B**) salicylic acid contents in the leaves of peppers grown under normal and stress conditions and treated with plant growth-promoting rhizobacteria (PGPR) after eight days. Treatment: Cont (control), PGPR (*Bacillus amyloliquefaciens*), BOT (*Botrytis pelargonii*), PGPR + BOT (*Bacillus amyloliquefaciens* + *Botrytis pelargonii*), ALT (*Alternaria alternata*), PGPR + ALT (*Bacillus amyloliquefaciens* + *Alternaria alternata*). Values show the means ± standard error (*n* = 3) and significant differences are indicated at *p* < 0.05 in accordance with the least significant difference test. Bars with different letters are significantly different from each other.

Plant hormone analysis showed the differential accumulation of ABA and SA in PGPR-inoculated pepper plants under control and biotic stress conditions over eight days. Biotic stresses caused increases in ABA in the pepper seedlings. The PGPR treatment decreased the ABA levels in pepper plants compared to those of control plants in the absence of biotic stress. Upon exposure to *Botrytis* and *Alternaria* stresses, PGPR-inoculated plants exhibited significantly reduced ABA content (73.82% and 67.74%, respectively) compared with those of non-inoculated stressed plants (Figure 5A).

In contrast to the stressed plants without treatment, non-inoculated plants displayed decreases in SA concentrations of 75.05% (*Botrytis*) and 76.52% (*Alternaria*), respectively, compared to those of the control plants. As shown in Figure 5B, seedlings inoculated with PGPR for eight days showed remarkable 74.68% and 68.30% increases in SA contents in pepper plants under *Botrytis* and *Alternaria* stresses compared with those of non-inoculated stressed plants. The results suggested that PGPR inoculation enhanced the SA content in pepper seedlings with or without stress.

### 3.7. Free Amino Acid Content

**Figure 6:**
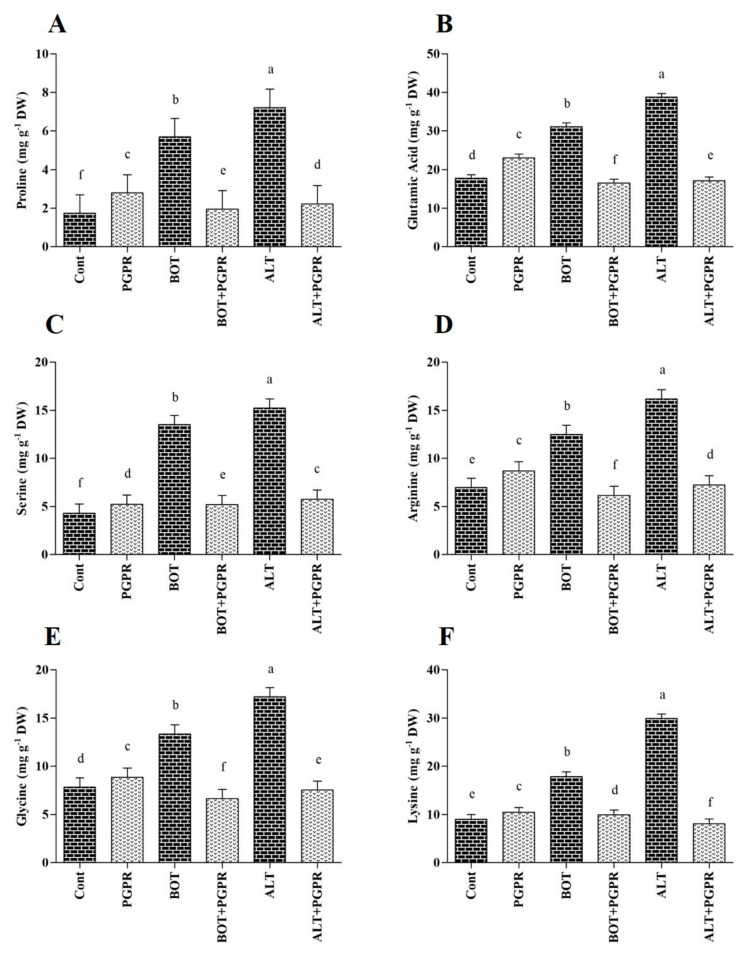
Amino acid contents ((**A**) Proline; (**B**) Glutamic acid; (**C**) Serine; (**D**) Arginine; (**E**) Glycine; (**F**) Lysine) in the leaves of peppers grown under normal and stress conditions and treated with plant growth-promoting rhizobacteria (PGPR) after eight days. Treatment: Cont (control), PGPR (*Bacillus amyloliquefaciens*), BOT (*Botrytis pelargonii*), PGPR + BOT (*Bacillus amyloliquefaciens* + *Botrytis pelargonii*), ALT (*Alternaria alternata*), PGPR + ALT (*Bacillus amyloliquefaciens* + *Alternaria alternata*). Values show the means ± standard error (*n* = 3) and significant differences are indicated at *p* < 0.05 in accordance with the least significant difference test. Bars with different letters are significantly different from each other.

Six amino acids were detected with different concentrations in pepper seedlings (Figure 6). Biotic stresses increased the amino acid content of the pepper seedlings compared to those under normal conditions over eight days. Proline (Pro) levels increased by 69.44% and 75.92% in *Botrytis-* and *Alternaria*-stressed plants, respectively. However, plants with PGPR treatment showed a decrease in Pro levels. Amino acid levels decreased eight days after the application of PGPR to stressed plants. For instance, Pro levels decreased by 65.83% and 69.36% in PGPR-treated plants under *Botrytis* and *Alternaria* stress, respectively, compared to those of stressed plants without treatment (Figure 6).

### 3.8. Soluble Protein and Sugar Contents

**Figure 7:**
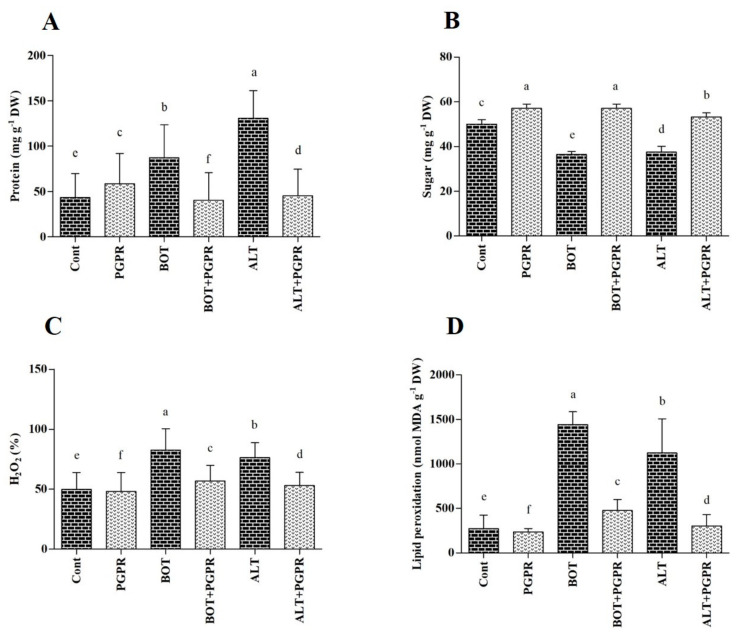
(**A**) protein, (**B**) sugar, (**C**) hydrogen peroxide, and (**D**) malondialdehyde contents in the leaves of peppers grown under normal and stress conditions and treated with plant growth-promoting rhizobacteria (PGPR) after eight days. Treatment: Cont (control), PGPR (*Bacillus amyloliquefaciens*), BOT (*Botrytis pelargonii*), PGPR + BOT (*Bacillus amyloliquefaciens* + *Botrytis pelargonii*), ALT (*Alternaria alternata*), PGPR + ALT (*Bacillus amyloliquefaciens* + *Alternaria alternata*). Values show the means ± standard error (*n* = 3) and significant differences are indicated at *p* < 0.05 in accordance with the least significant difference test. Bars with different letters are significantly different from each other.

The protein levels increased in diseased seedlings after eight days. In uninfected conditions, protein levels increased by 26.35% upon PGPR inoculation compared with plants non-inoculated with the bacterium. The protein production rates improved by 50.60% and 67.00% under *Botrytis* and *Alternaria* stresses, respectively, compared to those of non-stressed plants without treatment (*p* < 0.05) (Figure 7A). Conversely, a decrease in protein levels was detected in PGPR-treated plants when subjected to *Botrytis* (54.01%) and *Alternaria* (65.49%) stresses compared with those of plants subjected to biotic stresses.

A decrease in sugar levels occurred after exposure to pathogen attack (Figure 7B). As shown in Figure 7B, the sugar contents were alleviated in response to *Botrytis* (27.20%) and *Alternaria* (24.80%) stresses compared to those of plants under normal conditions. The optimum outcomes were obtained once plants were treated with PGPR, which contributed to an increase in sugar contents of 36.25% and 29.32% under *Botrytis* and *Alternaria* stress conditions, respectively, compared with those of untreated stressed plants (Figure 7B).

### 3.9. H_2_O_2_ and MDA Content

The H_2_O_2_ levels were assessed to determine whether PGPR application attenuated the effects of stress on pepper seedlings. Biotic stresses caused substantial modification in H_2_O_2_ contents in pepper plants (Figure 7C). The H_2_O_2_ levels increased by 39.52% and 34.66% under *Botrytis* and *Alternaria* stresses, respectively, compared to those of the control plants. The inoculation of PGPR successfully decreased H_2_O_2_ levels in stressed plants. The greatest decreases in H_2_O_2_ levels of 30.98% and 30.43% were noted in PGPR-inoculated plants under *Botrytis* and *Alternaria* stresses, respectively (*p* < 0.05).

As illustrated in Figure 7D, stress conditions led to the increase in MDA production in untreated pepper plants. The MDA levels increased by 81.05% and 75.71% under *Botrytis* and *Alternaria* stresses, respectively. Compared with the untreated plants, the decreases in MDA levels in the PGPR-treated plants were approximately 66.89% under *Botrytis* and 73.04% under *Alternaria* stress conditions (*p* < 0.05).

### 3.10. Antioxidant Content

**Figure 8:**
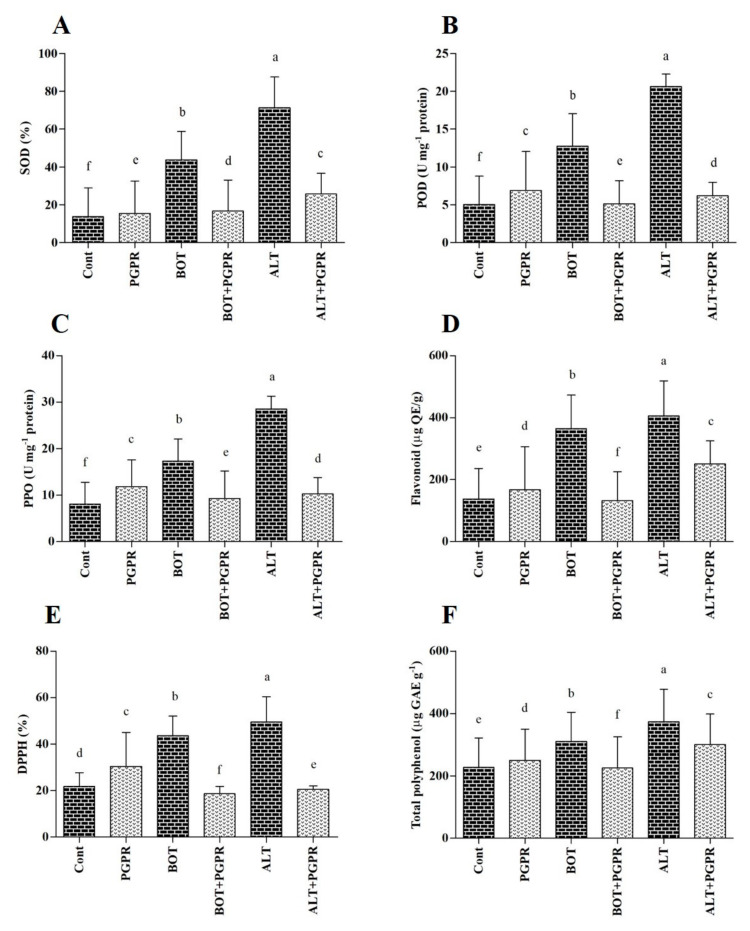
Antioxidant contents ((**A**), SOD; (**B**), peroxidase; (**C**), polyphenol oxidase; (**D**), flavonoid; (**E**), DPPH; (**F**), total polyphenol) in leaves of peppers grown under normal and stress conditions and treated with plant growth-promoting rhizobacteria (PGPR) after eight days. Treatment: Cont (control), PGPR (*Bacillus amyloliquefaciens*), BOT (*Botrytis pelargonii*), PGPR + BOT (*Bacillus amyloliquefaciens* + *Botrytis pelargonii*), ALT (*Alternaria alternata*), PGPR + ALT (*Bacillus amyloliquefaciens* + *Alternaria alternata*). Values show the means ± standard error (*n* = 3) and significant differences are indicated at *p* < 0.05 in accordance with the least significant difference test. Bars with different letters are significantly different from each other.

SOD activity augmentation occurred under stress conditions. SOD activity decreased by 61.96% and 64%, respectively, in PGPR-treated plants exposed to *Botrytis* and *Alternaria* stresses compared to those of untreated stressed plants (Figure 8A).

POD, PPO, and flavonoid activities followed similar patterns; their activities increased under stress conditions. PGPR treatment reduced POD, PPO, and flavonoid levels under stress conditions compared to those of untreated stressed plants. For instance, their activities decreased (POD, 59.77%; PPO, 46.21%; flavonoid, 63.67%) in PGPR-inoculated seedlings subjected to *Botrytis* stress (*p* < 0.05) (Figure 8B–D).

DPPH levels in pepper seedlings increased under stress, while they decreased in PGPR-treated seedlings exposed to biotic stress. PGPR inoculation assisted in lowering DPPH contents by 57.12% and 58.29% under *Botrytis* and *Alternaria* stresses, respectively, compared with those of untreated stressed plants (Figure 8E).

Total polyphenol levels increased slightly under stress conditions. On the other hand, these levels decreased upon PGPR inoculation under stress conditions (Figure 8F).

### 3.11. Nutrient Content in Plants

To determine the effects of the PGPR inoculant on the nutrient levels of pepper plants, three elements; Ca, K, and P, were examined (Table 4). In uninfected plants, increases were observed in the concentrations of K and P of plants inoculated with PGPR compared to those of control plants. Additionally, Ca levels increased in inoculated plants compared to those of uninfected control plants. Compared with the stressed plants and PGPR-inoculated plants, the inoculated infected plants showed increases in the concentrations of Ca, K, and P.

### 3.12. Effect of B. amyloliquefaciens Treatment on the Regulation of Biotic Stress Responsive Genes

The expression of biotic stress responsive genes was investigated in pepper seedlings. Overall, 12 genes (Table S3) were assessed for their change in expression under biotic stresses and PGPR application in pepper plant seedlings.

### 3.13. Antimicrobial and Defense-Related Protein (CaAMP1, CaPR1, and CaDEF1)

**Figure 9:**
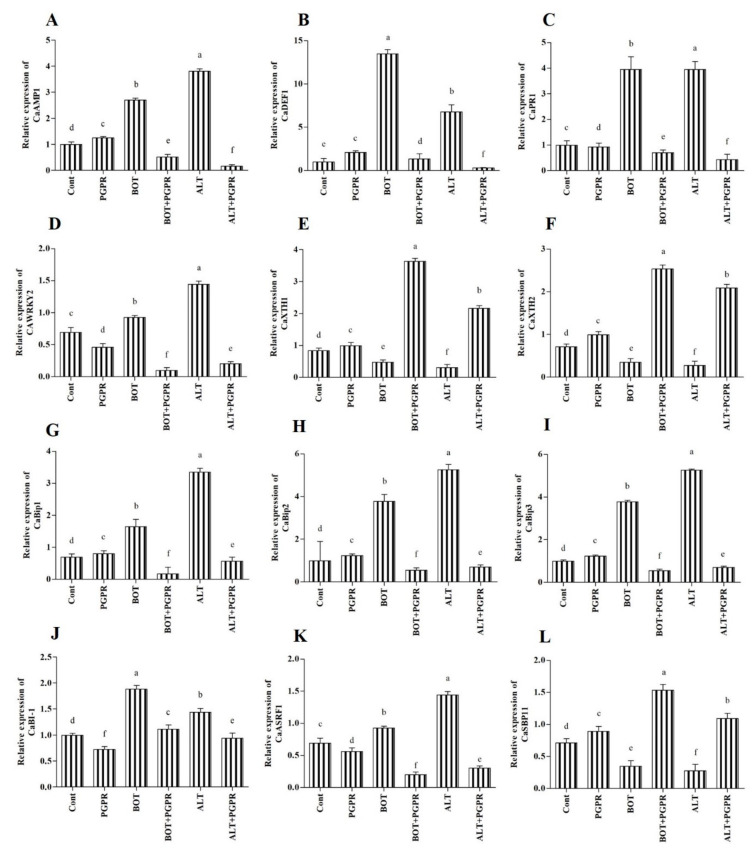
Real-time expression analysis of CaAMP1 (**A**), CaDEF1 (**B**), CaPR1 (**C**), CaWRKY2 (**D**), CaXTHs (CaXTH1; (**E**), and CaXTH2; (**F**)), CaBiPs (CaBiP1; (**G**), CaBiP2; (**H**), and CaBiP3; (**I**)), CaBI-1 (**J**), CaASRF1 (**K**), and CaSBP11 (**L**) in the leaves of peppers grown under normal and stress conditions and treated with plant growth-promoting rhizobacteria (PGPR) after eight days. Treatment: Cont (control), PGPR (*Bacillus amyloliquefaciens*), BOT (*Botrytis pelargonii*), PGPR + BOT (*Bacillus amyloliquefaciens* + *Botrytis pelargonii*), ALT (*Alternaria alternata*), PGPR + ALT (*Bacillus amyloliquefaciens* + *Alternaria alternata*). Values show the means ± standard error (*n* = 3) and significant differences are indicated at *p* < 0.05 in accordance with the least significant difference test. Bars with different letters are significantly different from each other.

The transcription pattern of CaAMP1, CaPR1, and CaDEF1 was evaluated in pepper plants treated with either biotic stresses or PGPR. As illustrated in Figure 9A–C, CaAMP1, CaPR1, and CaDEF1 levels increased considerably in diseased plants. For instance, infected plants registered higher CaAMP1 expression (63.15% under *Botrytis* and 73.87% under *Alternaria* stress conditions) compared to that of unstressed plants. In contrast, stressed plants inoculated with PGPR had lower CaAMP1, CaPR1, and CaDEF1 expression compared with that of untreated stressed plants. In reaction to PGPR treatment, CaAMP1 expression decreased by 80.93% and 95.91% in *Botrytis*- and *Alternaria*-infected plants, respectively.

### 3.14. Transcription Factor WRKY2

The expression levels of transcription factor WRKY2 (CaWRKY2) were examined. *Botrytis*- and *Alternaria*-stressed plants showed 89.77% and 79.57% increases in CaWRKY2 expression, respectively, compared to that of unstressed plants. However, PGPR-inoculated stressed plants showed a decrease in gene expression compared to that of the untreated stressed plants. CaWRKY2 expression in stressed pepper plants decreased in the PGPR-inoculated plants (89.05% under *Botrytis* and 85.94% under *Alternaria* stresses) compared to that of the untreated stressed plants (Figure 9D).

### 3.15. Xyloglucan Endotransglucosylase/Hydrolase (XTH)

The expressions of CaXTH1 and CaXTH2 genes in pepper seedlings under biotic stresses and PGPR inoculation are shown in Figure 9E,F. Analysis of CaXTH genes showed changes in the expression of PGPR-inoculated pepper plants under stress. Application of biotic stresses reduced CaXTH gene expression in pepper plants despite an increase in their expression in PGPR-treated plants. In particular, PGPR exposure enhanced CaXTH2 gene expression by approximately 86.29% and 86.84% under *Botrytis* and *Alternaria* stresses compared with the corresponding untreated stressed plants.

### 3.16. Binding Protein (BiP)

Three BiP genes (*CaBiP1*, *CaBiP2*, and *CaBiP3*) were identified in pepper plants. These genes revealed the distinct reactions of PGPR-inoculated pepper seedlings during biotic stress. The expression level of *CaBiP1* increased in stressed plants compared to that of the unstressed control plants. However, among the stressed plants, the PGPR-inoculated plants demonstrated 89.60% (*Botrytis*) and 83.08% (*Alternaria*) decreases in expression compared to that of the untreated plants (Figure 9G). Increased expression of the CaBiP2 gene was detected in the untreated stressed plants compared to that of the inoculated control plants. PGPR-inoculated plants had lower *CaBiP2* gene expression compared to that of the untreated plants during stress conditions (Figure 9H). Biotic stresses affected the expression of *CaBiP3* in pepper plants. The *CaBiP3* expression increased in stressed plants compared to that of the control plants. PGPR-inoculated *Botrytis*-stressed plants had 85.36% lower *CaBiP3* expression than that of the untreated salt-stressed plants. Under the *Alternaria* stress condition, *CaBiP3* expression decreased considerably (86.67%) in PGPR-inoculated stressed plants (Figure 9I).

### 3.17. BCL2-Associated x Protein (BAX) Inhibitor 1 (BI-1)

The expression of the BI-1 gene (CaBI-1 gene) under biotic stress was assessed in pepper seedlings that had been inoculated with PGPR (Figure 9J). We identified minor differences in expression between the control and PGPR-inoculated uninfected plants; higher expression was identified in stressed plants. PGPR-inoculated plants showed decreased BI-1 expression (40.89% under *Botrytis* and 34.76% under *Alternaria* stress conditions) compared to that of untreated stressed plants.

### 3.18. RING-Type E3 Ligases (ASRF1)

Enhanced CaASRF1 expression was observed in pepper seedlings subjected to biotic stress. The CaASRF1 expression level raised by 25.25% *(Botrytis)* and 51.98% *(Alternaria)* compared to the unstressed control plants (Figure 9K). However, PGPR-inoculated pepper plants showed reduced CaASRF1 expression under stress condition. *Botrytis*- and *Alternaria*-infected plants exhibited 78.26% and 79.01% lower CaASRF1 expression compared to the untreated stressed plants.

### 3.19. Squamosa Promoter Binding Protein (SBP)

The CaSBP11 expression level in pepper seedlings under biotic stresses and PGPR application is depicted in Figure 9L. Decrease in CaSBP11 expression level was detected in *Botrytis*- and *Alternaria*-stressed plants by 51.20% and 61.37%, respectively, as compared to the unstressed plants. On the other hand, application of PGPR increased the CaSBP11 expression content in *Botrytis* (77.36%) and *Alternaria* (74.79%) diseased pepper plants in comparison with the corresponding untreated stressed plants.

## 4. Discussion

During crop cultivation, biotic stress resulting from plant pathogens is a serious challenge that causes enormous economic losses for growers. Different agrochemicals are currently employed to control plant diseases. However, their usage is problematic due to public concern regarding dangerous residues, the selection of resistant strains of the pathogens, and increased expenses for plant protection. The development of microbe-based control methods could produce effective substitutes for managing crop disease. At present, free-living, nonpathogenic, root-colonizing bacteria are implemented in a broad range of agricultural production systems as bioinoculants in a variety of economically important plants [72,73].

Our study showed antagonistic activity of *B. amyloliquefaciens*, a plant growth promoting bacterium, against *Botrytis pelargonii* and *Alternaria alternata* under in vitro conditions. The activity was mediated by hydrolytic enzyme activity. The influence of this interaction was apparent under in vivo conditions for diminished fungal diseases. Inoculation with *B. amyloliquefaciens* resulted in increased growth and improved health of the pepper plants with or without *Botrytis pelargonii* and *Alternaria alternata* infection. Augmentation in the chlorophyll and carotenoid contents was observed in the leaves of PGPR-inoculated plants exposed to stress conditions. In addition, we found that the treatment with *B. amyloliquefaciens* increased Ca, K, and P levels compared to non-inoclulated plants. The increases in Ca levels are known to increase resistance to fungal infections in many crops [74,75]. Inoculation with plant growth promoting rhizobacteria (PGPRs) inoculation enhances the photosynthetic pigments in plants during stress conditions [76,77]. This corresponds to improved nutrient uptake from the rhizosphere, which sustains plant growth under stressful conditions [78,79]. It should be noted that inoculation with *B. amyloliquefaciens* improved the soil moisture level in diseased and healthy pepper plants. In agreement with our results, previous studies indicated that PGPRs promote soil moisture content, thus enhancing plant growth and survival [80,81]. This could be due to the formation of exoploysaccharides and biofilm by PGPRs [80].

Xyloglucan endotransglucosylase/hydrolase (XTHs) are responsible for the regulation of various physiological processes, including the elongation of plant cells [82]. Furthermore, they modulate plant response to environmental stimuli, including salinity, water deficit, and heat [83,84,85]. Our results revealed reduced XTH expressions in diseased plants. This could be due to fungal attack mechanisms, which causes decreased cell wall extensibility and growth reduction of seedlings. Muñoz-Bertomeu and Lorences [86] showed that the expressions of various XTHs decrease as pathogen infection progresses. PGPR-inoculated plants showed higher XTH expressions under pathogen attack, which induced improved plant height and leaf area. Overexpression of XTHs enhances plant tolerance toward abiotic stresses [84,87]. Our results confirm that the high expression of XTHs (CaXTH1 and CaXTH2) is associated with plant maintenance and tolerance in diseased pepper seedlings without unfavorable effects.

Phytohormones synergistically or antagonistically function in a complicated network to modulate multiple facets of plant growth, reproduction, and immunity [88]. Salicylic acid (SA) contributes to the photosynthetic and growth parameters as well as antagonized oxidative damage in plants in response to natural attackers [89,90]. Melotto et al. [91] showed antagonistic interactions between SA and abscisic acid (ABA) signaling in response to pathogens. Our results showed that pathogen stress increased ABA levels but decreased SA levels, which is in agreement with the results of previous studies [92,93]. These results indicate that PGPR application relieves pathogen stress in pepper seedlings by decreasing their ABA levels and increasing their SA levels.

Protein post-translational modification events, such as ubiquitination, have been detected during plant stress responses, growth, and development [94]. It has been proven that various ABA signaling convertors are exposed to the modulation via ubiquitination. Several kinds of E3 ligases have been recognized that modulate ubiquitination of ABA receptors [94]. Joo et al. [95] indicated that CaASRF1 gene (*Capsicum annuum* ABA Sensitive RING Finger E3 ligase 1) assuredly regulates ABA signaling pathway and plant development. They found that CaASRF1 gene alters drought stress endurance via ABA signaling. Our findings showed enhanced CaASRF1 expression in diseased plants, which was along with boosted ABA level. This confirmed that CaASRF1 gene certainly regulate ABA signaling and biotic stress response in diseased pepper plants.

SBP-box genes (SBP) have a crucial role in plant development, signal conduction, and reaction to abiotic and biotic stresses [96,97]. Previous study reported that SBP5 improved resistance to *Erysiphe necator* through SA disease resistance signaling mechanisms [98]. In our study, reduction in CaSBP11 level was detected in infected plants. In contrast, PGPR-inoculated plants showed enhanced CaSBP11 expression under *Botrytis* and *Alternaria* attack. Zhang et al. [99] confirmed that CaSBP11 increased pepper plant defense response to *Phythophthora capsici* by regulating SA and jasmonic acid signaling mechanisms. Based on the obtained results, it is speculated that CaSBP11 positively modulates SA signaling pathways to enhance disease resistance in *Botrytis*- and *Alternaria*-infected plants.

WRKY, a major transcription factor family, was found to be involved in numerous developmental and physiological functions comprising abiotic and biotic stress signaling pathways [100,101,102]. WRKY expression increased upon pathogen stress conditions, which was consistent with the results of a previous study [103]. Additionally, a number of WRKY transcription factors act in ABA and SA signaling pathways. Xie et al. [104] found that ABA either negatively or positively modulates the transcripts of some WRKYs. We found that in the presence of ABA, WRKY2 expression increased in diseased pepper plants. These results indicated that ABA positively mediates the expression of WRKY2. Upon applying PGPR, the SA level increased and the ABA and WRKY2 levels decreased under stress condition. Dong et al. [105] found that upon stimulating the SA-dependent defense, WRKYs revealed various modulations comprised of suppression or augmentation. These results indicated that PGPR application allows the stressed plants to face various biotic stresses.

Adverse environmental cues have a negative influence on plant growth and development and induce protein denaturation or misfolding [106,107]. Endoplasmic reticulum stress is triggered by misfolded proteins that accumulate in the endoplasmic reticulum under harmful environmental situation and lead to programmed cell death [108]. Misfolded or unfolded protein augmentation in endoplasmic reticulum has impact on cellular protein function and localization [109]. BiPs play a crucial role in protein quality monitoring by distinguishing and refolding misfolded proteins. Furthermore, by alleviating the amount of unfolded protein, BiPs balance immune receptors to ease plant defense [110]. In this study, biochemical and molecular approaches identified an increase in the protein value of diseased plants (Figure 3A and Figure 5A–C). Protein augmentation caused BiP genes (*CaBiP1*, *CaBiP2*, and *CaBiP3*) induction in *Botrytis* and *Alternaria*-infected plants. It has been shown that the aggregation of unfolded proteins promotes BiP induction to ameliorate plant endurance to abiotic and biotic stresses [109]. In contrast, BiPs were restrained in PGPR-inoculated plants exposed to pathogen stress. Together, these results suggest that *B. amyloliquefaciens* mitigates the soluble protein content in diseased plants. This might be due to the stress-soothing effect of this PGPR, which results in protein catabolism.

Plant exposure to biotic and abiotic stresses leads to the generation of reactive oxygen species (ROS) to trigger the stress response and defense pathways. ROS overaccumulation causes oxidative damage, impaired membrane lipid functions, enzyme inactivation, impeded metabolic activities, and, ultimately, plant death [111]. We found that H_2_O_2_ and MDA levels increased in infected plants, which was in accordance with the results of previous studies [111]. PGPR-inoculated plants showed lower H_2_O_2_ and MDA levels when under pathogen attack. This suggested that PGPR application might restrain the production of ROS, successfully inhibiting cell damage under oxidative stress conditions [112,113,114].

ROS are key players in programmed cell death (PCD); a cell suicide process that inhibits the pathogen spread in plants and eliminates damaged cells. BAX is a pivotal modulator of PCD and is stabilized by the function of the anti-PCD factor BI-1 [115]. BI-1 demonstrates inducement as opposed to numerous types of biotic and abiotic environmental stresses and provides endurance in plants against these stressors [116,117,118]. Increased expression of CaBI-1 was observed in diseased pepper plants. CaBI-1 expression was decreased in PGPR-inoculated plants exposed to pathogen invasion. This validated the potency and attenuating influence of PGPR in pepper plants affected by *Alternaria* or *Botrytis* stress.

Plants have defensive mechanisms comprised of antioxidants with enzymatic or non-enzymatic activity to survive oxidative damage and neutralize immoderate oxidation [119]. PGPR increases the activity of enzymatic/non-enzymatic antioxidants. Consistently, our results have validated the amelioration of antioxidant activity in healthy plants. In the current study, the activity of antioxidants increased in the diseased plants, whereas their activity decreased in PGPR-inoculated plants subjected to *Botrytis* or *Alternaria* stress. This decreased activity implies progress in scavenging over accumulated ROS and minimizing oxidative damage, which maintains optimal protection of the plant [119].

Environmental stress lowers leaf sugar content, leading to physiological and biochemical modifications as sugar sustains macromolecules and membrane structure during stress [120]. Accumulated soluble sugars can induce pathogen resistance in plants as the soluble sugars or sugar byproducts can play a role as osmoprotectants under stress conditions [121]. In the present study, an increase in leaf sugar content was identified in PGPR-inoculated plants under normal as well as stress conditions. Soluble sugars function as metabolic resources and structural constituents of cells and modulate many processes connected with plant development under stress conditions [122]. Sugar accumulation in leaves also triggers the expression of genes connected to photosynthetic activities [123]. In this study, PGPR triggered major sugar accumulation, which potentially acted as an osmoprotectant in the photosynthetic organs and assisted with the retention of photosynthetic performance, leading to improved growth and defense mechanisms under *Botrytis* and *Alternaria* attack.

Amino acids act as precursors for metabolite synthesis and modulate plant responses to environmental stress [124]. Our results showed an increase in amino acid contents in infected plants. This augmentation indicates that they play a role in plant defense in addition to their roles in metabolism [125,126]. Previous studies have indicated that amino acid levels increase under pathogen stress [126,127]. In the present study, the PGPR treatment decreased the amino acid level in *Botrytis*- and *Alternaria*-stressed plants. The accumulation of Pro, which acts as an osmolyte and ROS scavenger, could be a strategy for withstanding pathogen invasion [127,128]. The reduced Pro content in PGPR-inoculated plants might be caused by osmotic adjustment, which leads to improved plant survival under pathogen attack.

Plants trigger a series of responses toward microbial attacks, which induce a range of antimicrobial defenses both locally and systematically [129]. These defense responses include the strengthening of mechanical barriers, oxidative burst, and the production of antimicrobial and defensive compounds [130,131]. The antimicrobial protein gene CaAMP1 was strongly induced in infected pepper plants to enhance tolerance to fungal disease, which is in agreement with the results of previous studies [132,133,134,135]. AMPs play a crucial role in constitutive or triggered tolerance to various pathogens by deteriorating fungal cell walls, stimulating membrane channels, and preventing DNA synthesis [134,136]. Moreover, infection of *Botrytis* and *Alternaria* spp. highly activated the expression of defense-related genes; namely, CaDEF1 and CaPR1, in the pepper plants. Previous studies have demonstrated the expression of defense-related genes in pepper and tomato plants upon infection with pathogens, such as *Phytophthora capsici*, *Xanthomonas campestris*, and *Clavibacter capsici* [137,138]. In contrast, CaAMP1, CaDEF1, and CaPR1 expressions were strongly decreased in PGPR-inoculated plants subjected to biotic stress. Together, these results confirm the involvement of CaAMP1, CaDEF1, and CaPR1 in resistance to fungal pathogens and demonstrated the stress-relieving effect of PGPR.

## 5. Conclusions

Along with enhancing pepper growth, the bacterium *B. amyloliquefaciens* stimulates resistance to *Botrytis pelargonii* and *Alternaria alternata* infections. This bacterial strain can secrete hydrolytic enzymes and solubilize nutrients, thus promoting host growth and alleviating the disease in the pepper plant. Additionally, PGPR treatment influences the host biochemistry increasing resistance to the infections by fungal pathogens. PGPR application triggered the expression of stress-related genes, namely, CaAMP1, CaDEF1, CaPR1, CaXTH, CaWRKY2, CaBI-1, CaASRF1, CaSBP11, and CaBiP. This study is the first report, to our knowledge, of the suppressive effects of *B. amyloliquefaciens* on *Alternaria* leaf spot and *Botrytis* gray mold of *C. annum*. The results of this study indicate that *B*. *amyloliquefaciens* is beneficial for *C. annum* under biotic stress and may be suitable as a candidate for the management of crop diseases.

## Figures and Tables

**Table 1:** Experimental work plan.

Symbol	Treatment
Cont	treated with sterile distilled water
PGPR	treated with PGPR
BOT	treated with BOT
BOT+PGPR	treated with BOT + PGPR
ALT	treated with ALT
ALT + PGPR	treated with ALT + PGPR

Cont: Control; PGPR: *Bacillus amyloliquefaciens*; BOT: Botrytis pelargonii; ALT: *Alternaria alternate*.

**Table 2:** Hydrolytic enzyme activity of bacterial strain and its effect on the inhibition of *Botrytis pelargonii* and *Alternaria alternata* growth.

Bacterial Isolate	Isolated Host	Accession No.	Hydrolytic Enzyme Production									Inhibition (mm)	
			Amylase	Protease	Pectinase	Cellulase	Lipase	Catalase	Glucanase	Laccase	Phytase	ALT	BOT
*Bacillus amyloliquefaciens*	*Sasamorpha borealis*	MW599955	+	+	+	+	+	+	+	+	+	65.66 ± 1.0	69.50 ± 0.5

+ indicates a positive response.

**Table 3:** Effect of plant growth-promoting rhizobacteria (PGPR) inoculation on pepper plant growth as well as chlorophyll a (Chla), chlorophyll b (Chlb), total chlorophyll (total Chl), and carotenoid contents under normal and stress conditions after eight days.

Treatment	Plant Height	Stem Diameter	Leaf Length	Leaf Width	Total Plant Fresh Weight	Chla	Chlb	Total Chl	Carotenoid	No. Leaf
	(cm)	(cm)	(cm)	(cm)	(g)	µg/g FW	µg/g FW	(µg/g FW)	µg/g FW	
8DAT										
Cont	20.3 ± 0.3 ^c^	0.3 ± 0.02 ^a^	9.1 ±0.03 ^c^	5.3 ± 0.2 ^d^	12.0 ± 0.05 ^c^	21.8 ± 7.2 ^e^	25.9 ± 2.5 ^b^	107.63 ± 1.7 ^d^	1.0 ± 0.2 ^d^	16.0 ± 0.0 ^b^
PGPR	20.5 ± 0.1 ^b^	0.3 ± 0.05 ^a^	10.7 ± 0.3 ^a^	6.6 ± 0.1 ^a^	16.2 ± 0.06 ^a^	24.2 ± 6.0 ^c^	28.0 ± 7.1 ^a^	118.7 ± 1.6 ^c^	1.2 ± 0.5 ^c^	17.6 ± 0.3 ^a^
BOT	14.6 ± 0.3 ^f^	0.2 ± 0.002 ^b^	7.6 ± 0.5 ^d^	4.0 ± 0.2 ^e^	7.95 ± 0.15 ^f^	22.1 ± 2.9 ^d^	12.5 ± 0.7 ^d^	96.9 ± 1.3 ^e^	0.8 ± 3.1 ^e^	13.3 ± 0.0 ^e^
BOT + PGPR	21.6 ± 0.1 ^a^	0.3 ± 0.01 ^a^	9.5 ± 0.3 ^b^	5.7 ± 0.1 ^b^	12.20 ± 0.20 ^b^	34.9 ± 7.6 ^b^	13.2 ± 1.0 ^c^	140.58 ± 1.7 ^b^	1.9 ± 2.3 ^a^	16.0 ± 0.0 ^b^
ALT	15.8 ± 0.4 ^e^	0.2 ± 0.01 ^b^	6.6 ± 0.1 ^e^	3.8 ± 0.1 ^f^	8.30 ± 0.30 ^e^	15.9 ± 1.4 ^f^	6.0 ± 2.0 ^f^	64.21 ± 4.4 ^f^	0.6 ± 3.5 ^f^	15.0 ± 0.5 ^d^
ALT + PGPR	19.3 ± 0.3 ^d^	0.3 ± 0.008 ^a^	9.1 ± 0.2 ^c^	5.5 ± 0.1 ^c^	11.25 ± 0.25 ^d^	39.2 ± 7.9 ^a^	10.1 ± 1.3 ^e^	152.46 ± 8.4 ^a^	1.8 ± 3.8 ^b^	15.6 ± 0.3 ^c^

Treatment: Cont (control), PGPR (*Bacillus amyloliquefaciens*), BOT (*Botrytis pelargonii*), PGPR + BOT (*Bacillus amyloliquefaciens* + *Botrytis pelargonii*), ALT (*Alternaria alternata*), PGPR + ALT (*Bacillus amyloliquefaciens* + *Alternaria alternata*). Values show the means ± standard error (*n* = 3) and significant differences are indicated at *p* < 0.05 in accordance with the least significant difference test. Data within the same column followed by different letters are significantly different.

**Table 4:** Nutrient accumulation in pepper plants grown under biotic stress and control conditions treated with or without plant growth-promoting rhizobacteria.

Sample Name	Ca (ug/kg)	K (ug/kg)	P (ug/kg)
8DAT-Plant			
Cont	6.45 ± 0.05 ^d^	43.31 ± 1.31 ^d^	5.01 ± 0.01 ^d^
PGPR	8.38 ± 0.18 ^a^	49.64 ± 0.5 ^c^	6.76 ± 0.23 ^b^
BOT	6.15 ± 0.15 ^e^	43.01 ± 1.0 ^e^	4.66 ± 0.26 ^e^
BOT+PGPR	7.50 ± 0.20 ^b^	53.14 ± 0.86 ^a^	5.96 ± 0.04 ^c^
ALT	5.93 ± 0.13 ^f^	41.38 ± 0.58 ^f^	4.43 ± 0.23 ^f^
ALT + PGPR	6.94 ± 0.05 ^c^	52.70 ± 0.30 ^b^	6.95 ± 0.04 ^a^

Treatment: Cont (control), PGPR (*Bacillus amyloliquefaciens*), BOT (*Botrytis pelargonii*), PGPR + BOT (*Bacillus amyloliquefaciens* + *Botrytis pelargonii*), ALT (*Alternaria alternata*), PGPR + ALT (*Bacillus amyloliquefaciens* + *Alternaria alternata*). Values show the means ± standard error (*n* = 3) and significant differences are indicated at *p* < 0.05 in accordance with the least significant difference test. Data within the same column followed by different letters are significantly different.

## Data Availability

Not applicable.

## Supplementary Materials

The following are available online at https://www.mdpi.com/article/10.3390/jof7060472/s1, Figure S1: Symptomatic pepper plant collected from the farm, Figure S2: Effect of plant growth-promoting rhizobacteria (PGPR) inoculation on the pepper plants grown under normal and biotic stress conditions after eight days, Table S1: Primers used in this study for PCR amplification of the 18S rDNA fungal isolates, Table S2: Physiochemical properties of the soil samples over eight days of treatment, Table S3: Primers used for relative gene expression analysis.
