# Supplementary material for: Biocontrol Potential of Bacillus amyloliquefaciens against Botrytis pelargonii and Alternaria alternata on Capsicum annuum
Source: J Fungi (Basel). 2021 Jun 10;7(6):472. doi: 10.3390/jof7060472 (PMC8230671; doi:10.3390/jof7060472)
Supplement: Supplementary file 1 [file jof-07-00472-s001.zip › jof-1245185-supplementary.pdf]

Supplementary materials

Biocontrol potential of *Bacillus amyloliquefaciens* against *Botrytis pelargonii* and *Alternaria alternata* associated with *Capsicum annuum*

Elham Ahmed Kazerooni[1*], Sajeewa S. N. Maharachchikumbura[2], Abdullah Mohammed Al-Sadi[3], Sang-Mo Kang[1], Byung-Wook Yun[1], In-Jung Lee[1*]

[1]Department of Applied Biosciences, Kyungpook National University, Daegu 41566, Republic of Korea

[2]School of Life Science and Technology, University of Electronic Science and Technology of China, Chengdu 15 611731, People's Republic of China

[3]Department of Plant Sciences, College of Agricultural and Marine Sciences, Sultan Qaboos University, PO Box 34, Al-Khod 123, Oman

[*]Corresponding authors

Prof. In-Jung lee
Dr. Elham Ahmed Kazerooni
Crop Physiology Laboratory
Department of Applied Biosciences
Kyungpook National University
Daegu 41566, Republic of Korea
E-mail: ijlee@knu.ac.kr
E-mail: elham.ghasemi.k@gmail.com
Tel: +82-53-950-5708(Office)

Table S1. Primers used in this study for PCR amplification of the 18S rDNA fungal isolates

**Gene symbol**	**Primers (5′–3′) Forward/reverse**
ITS1	5′-TCC GTA GGT GAA CCT GCG G-3′
ITS4	5′-TCCTCCGCTTATTGATATGC-3′
RPB2-5F2	5′-GGGGWGAYCAGAAGAAGGC-3′
fRPB2-7cR	5′-CCCATRGCTTGYTTRCCCAT-3′
Alt-for	5’ATGCAGTTCACCACCATCGC’3
Alt-rev	5’ACGAGGGTGAYGTAGGCGTC’3
gpd 1	5’CAACGGCTTCGGTCGCATTG’3
gpd 2	5’GCCAAGCAGTTGGTTGTGC’3

Table S2. Physiochemical properties of the soil samples over eight days of treatment.

Sample name	Soil texture	pH	EC (mS)	Moisture (%)
8DAT				
Cont	sandy loam	7.0±0.0c	1.18±0.0d	70±0.0d
PGPR	sandy loam	7.9±1.0a	2.96±5.4a	86.6±11.5b
BOT	sandy loam	7.0±0.0c	0.4±0.0e	46.6±4.4e
BOT+PGPR	sandy loam	7.8±0.0b	2.1±0.0c	83.3±8.8c
ALT	sandy loam	6.9±6.2d	0.37±3.9f	45.0±5.7f
ALT+PGPR	sandy loam	7.9±6.2a	2.64±0.0b	90.0±10.0a

Values show the means ± standard error (n = 3) and significant differences at $p < 0.05$ in accordance with the least significant difference test.

Table S3. Primers used for relative gene expression analysis.

Gene symbol	Primers (5′–3′) Forward/reverse
CaWRKY2	5′-GTTTACCAATCGCCGAGACAG-3′<br>5′-CAGGTTCGTCACACTGCTCC-3′
CaBI-1	5′-ATATGGATCCATGGAGGGTTTCACGGTCGT-3′<br>5′-ATATGGATCCCTAGTTTCTCCTCTTCTTCTTC-3′
CaBiP1	5′-AGAGATCCCTCAGTAGCCAGC-3′<br>5′-GTTGTTCAACTCCTCAAAACGT-3′
CaBiP2	5′-AAGAAGTTGAGGCAGTGTGC-3′<br>5′-TGTGAATCGTCATCATCGTTG-3′
CaBiP3	5′-CAACATACTCTTGTGTGGGCG-3′<br>5′-TGAAGGGGTGATTCTGTTTCC-3′
CaXTH1	5′-ATCCCATTTCATCTTCAAATTAAAGC-3′<br>5′-GGGGAAATGATTTATTGTTATTTCG-3′
CaXTH2	5′-CTATGCCCGGCAGCTTGGGCTGAA-3′<br>5′-GACAACATTAGTAAACTCAATCC-3′
CaAMP1	5′-GAATTCATGGTTTCCAAAAGTAGTATTTTT-3′<br>5′-CTCGAGTTAGGCACAACAATAGTCACAACG-3′
CaPR1	5′-CAGGATGCAACACTCTGGTGG-3′<br>5′-ATCAAAGGCCGGTTGGTC-3′
CaDEF1	5′-CAAGGGAGTATGTGCTAGTGAGAC-3′<br>5′-TGCACAGCACTATCATTGCATAC-3′
CaASRF1	5′-ATGGGCCTCTCACAATATCCAACT-3′<br>5′-TCACATTGGACACGTATCGTCCTCT-3′
CaSBP11	5′-CGGGATCCATGGAGTCTTGGAGTTATTTCTCAGG-3′<br>5′-TCCCCCGGGGCAGTGATTCTAAGGCCGGG-3′
Actin	5′ -ACTCTTAATCAATCCCTCCACC-3′<br>5′-CTGTATGACTGACACCATCACC-3′

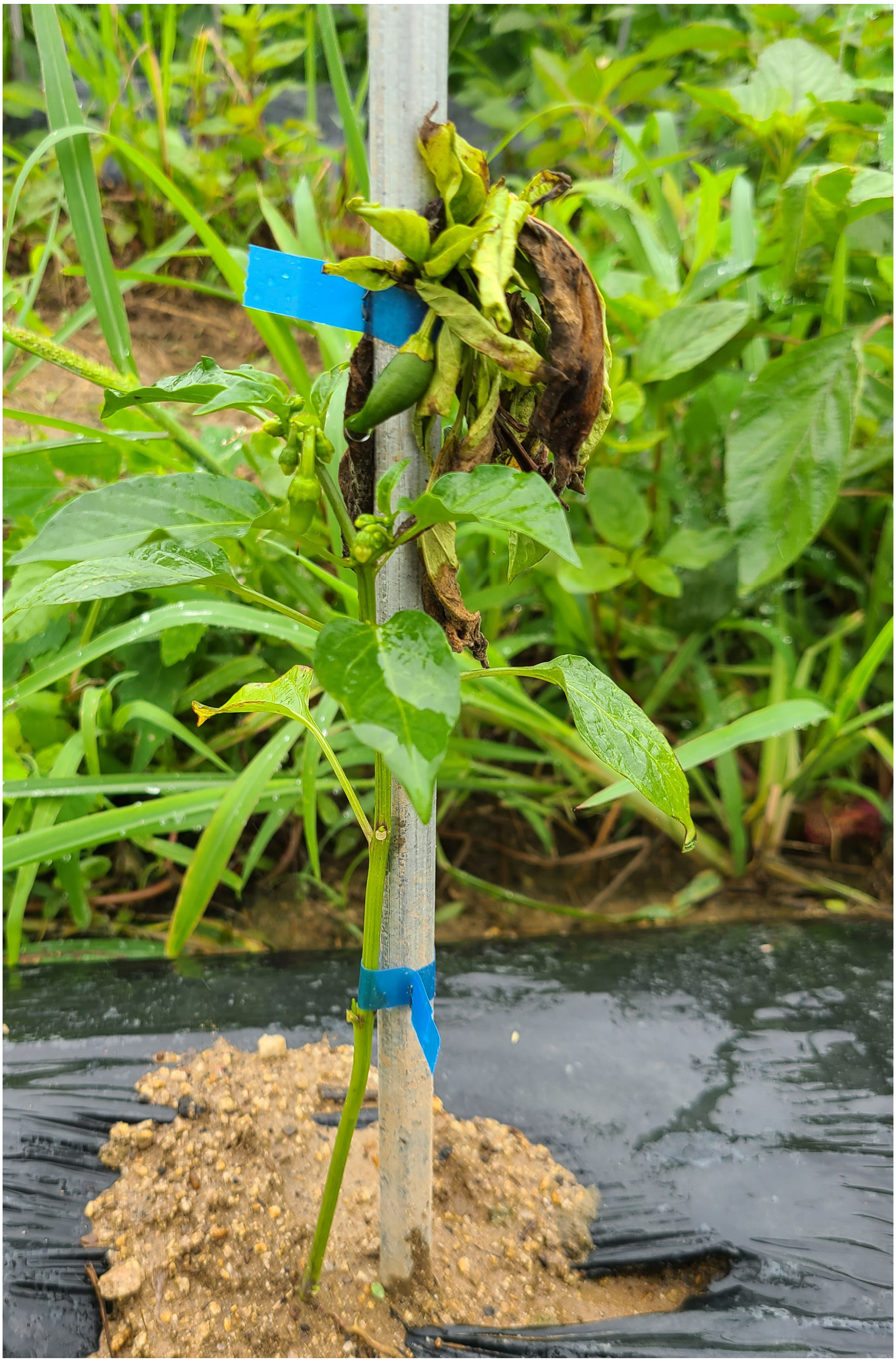

Figure S1. Symptomatic pepper plant collected from the farm

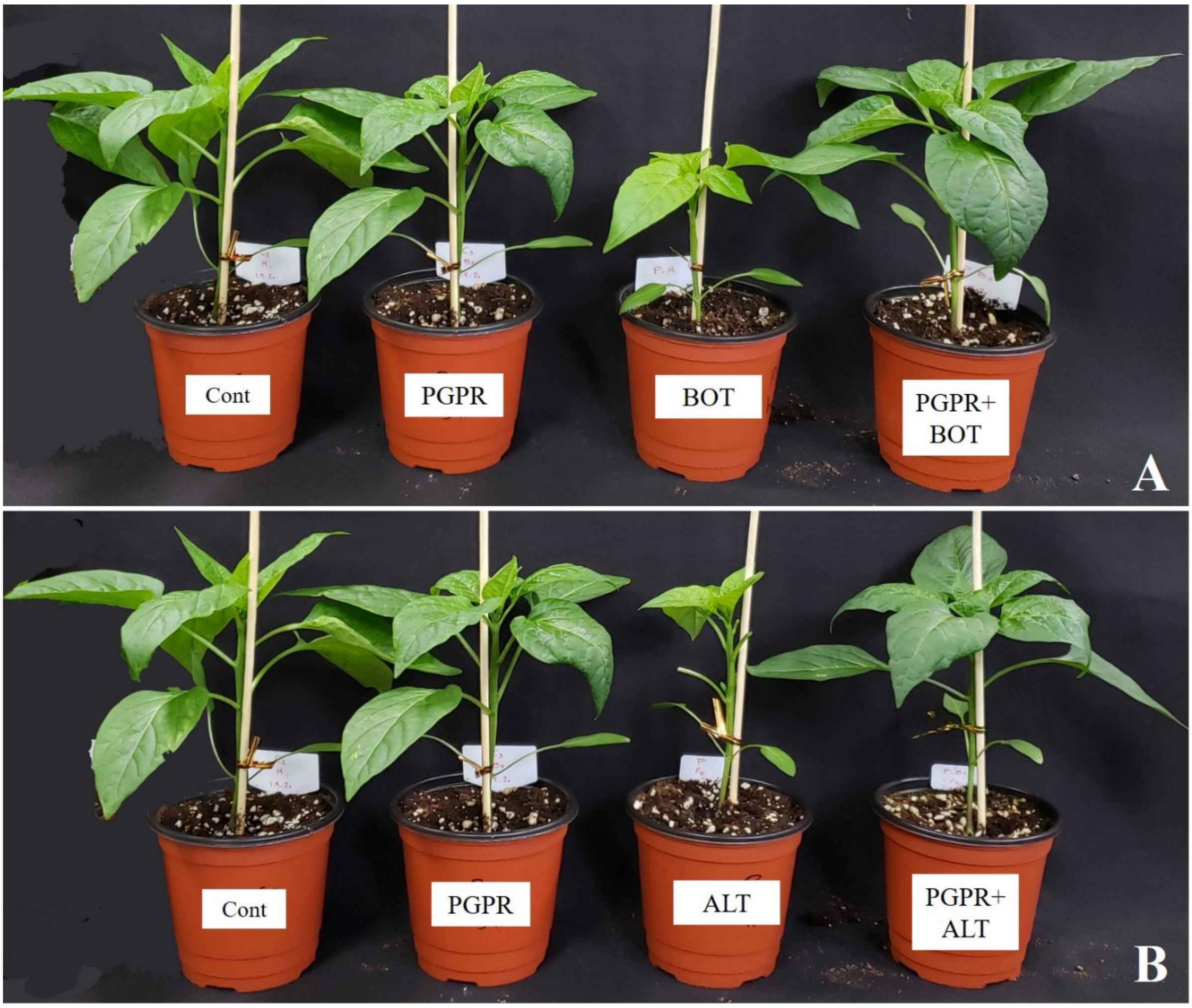

Figure S2. Effect of plant growth-promoting rhizobacteria (PGPR) inoculation on the pepper plants grown under normal and biotic stress conditions after eight days (A and B). Treatment: control, PGPR, BOT, PGPR + BOT, ALT, and PGPR + ALT.
